# Association between uric acid and the risk of hemorrhagic transformation in patients with acute ischemic stroke: a systematic review and meta-analysis

**DOI:** 10.3389/fneur.2024.1378912

**Published:** 2024-07-25

**Authors:** Ying Qian, Na Li, Yuanyuan Li, Chenxi Tao, Zhenhong Liu, Guoxia Zhang, Fan Yang, Hongrui Zhang, Yonghong Gao

**Affiliations:** ^1^Key Laboratory of Chinese Internal Medicine of Ministry of Education and Beijing, Dongzhimen Hospital, Beijing University of Chinese Medicine, Beijing, China; ^2^Institute for Brain Disorders, Beijing University of Chinese Medicine, Beijing, China; ^3^Guang'anmen Hospital, China Academy of Chinese Medical Sciences, Beijing, China

**Keywords:** acute ischemic stroke, uric acid, hemorrhagic transformation, correlation, meta-analysis

## Abstract

**Background:**

The relationship between hemorrhagic transformation (HT) and uric acid (UA) remains controversial. This study aimed to investigate the relationship between UA concentrations and the risk of HT following acute ischemic stroke (AIS).

**Methods:**

Electronic databases were searched for studies on HT and UA from inception to October 31, 2023. Two researchers independently reviewed the studies for inclusion. STATA Software 16.0 was used to compute the standardized mean difference (SMD) and 95% confidence interval (CI) for the pooled and post-outlier outcomes. Heterogeneity was evaluated using the I^2^ statistic and the Galbraith plot. Additionally, sensitivity analysis was performed. Lastly, Begg’s funnel plot and Egger’s test were used to assess publication bias.

**Results:**

A total of 11 studies involving 4,608 patients were included in the meta-analysis. The pooled SMD forest plot (SMD = −0.313, 95% CI = −0.586–−0.039, *p* = 0.025) displayed that low UA concentrations were linked to a higher risk of HT in post-AIS patients. However, heterogeneity (I^2^ = 89.8%, *p* < 0.001) was high among the studies. Six papers fell outside the Galbraith plot regression line, and there exclusive resulted in the absence of heterogeneity (I^2^ = 52.1%, *p* = 0.080). Meanwhile, repeated SMD analysis (SMD = −0.517, 95% CI = −0.748–−0.285, *p* = 0.000) demonstrated that the HT group had lower UA concentrations. Finally, Begg’s funnel plot and Egger’s test indicated the absence of publication bias in our meta-analysis.

**Conclusion:**

This meta-analysis illustrated a substantial connection between UA concentrations and HT, with lower UA concentrations independently linked with a higher risk of HT post-AIS. These results lay a theoretical reference for future studies.

**Systematic review registration:**https://www.crd.york.ac.uk/PROSPERO/CRD42023485539.

## Introduction

Acute ischemic stroke (AIS) is a primary contributor to mortality and long-term disabilities globally (1–3). This condition can be effectively managed with thrombolytic therapy (IVT) or endovascular thrombectomy (EVT) (4–7). Hemorrhagic transformation (HT) (8–10) can occur either as a natural progression of AIS or as a complication resulting from AIS treatment. Notably, it can elicit severe neurological deterioration, and even minor instances can adversely affect long-term functional outcomes. As a result, there is a pressing need to expand our understanding of HT to enhance the prognosis of AIS patients.

As reported by the Heidelberg criteria and the European Cooperative Acute Stroke Study classification (11–13), HT can be classified as either parenchymal hematoma (PH) or hemorrhagic infarction (HI); it can likewise be classified as asymptomatic intracranial hemorrhage (ICH) or symptomatic ICH (sICH), depending upon the presence of neurological deficits. Numerous risk factors (14–16) associated with the event of HT have been identified, including age, stroke severity, hyperglycemia, hypertension, levels of blood calcium and uric acid, and cholesterol levels. The increase in blood–brain barrier (BBB) permeability caused by inflammatory processes and free radical release is one of the chief causes of HT (3, 10, 17, 18).

Uric acid (UA) is derived from the metabolism of purines (19–21). Previous studies (22, 23) have illustrated that excessive UA concentrations are linked to an elevated risk of several disorders, including hypertension, chronic renal disease, cardiovascular and cerebrovascular diseases, etc. According to earlier studies (24, 25), UA concentrations are an independent risk factor for early mortality in AIS patients. It is the most substantial endogenous antioxidant (26–28), as reported by an increasing number of studies. It exerts neuroprotective effects by scavenging free radicals, suppressing the inflammatory cascade, and reducing BBB permeability. For instance, Lei et al. (29) and Wang et al. (19) discovered that UA may exert a protective effect on neurological outcomes post-AIS. Nonetheless, the relationship between UA and AIS prognosis remains unclear. While several studies have investigated the relationship between UA concentrations and HT following AIS, a definitive conclusion has not been reached. Thus, a meta-analysis was conducted to evaluate the correlation between UA concentrations and the risk of HT post-AIS.

## Methods

This meta-analysis was performed and reported following a predefined protocol (PROSPERO registration number: CRD42023485539) and the Preferred Reporting Items for Systematic Reviews and Meta-Analyses (PRISMA) guidelines.

### Search strategy

Databases such as PubMed, Cochrane Library, Embase, Web of Science, CBM (Chinese Biomedical Literature Database), CNKI (China National Knowledge Infrastructure), and Wanfang database were thoroughly and meticulously searched for relevant articles from inception to October 31,2023. The search terms included (“uric acid” OR “UA” OR “urate” OR “hyperuricemia”) AND (“Acute Ischemic Stroke” OR “AIS” OR “Ischemic Stroke” OR “Stroke”) AND (“hemorrhagic transformation” OR “HT”). Any additional articles identified were screened to expand the scope of the search. Given that all collected data were secondary summary data, the requirement for ethical approval was waived.

### Inclusion and exclusion criteria

Studies were individually screened by two reviewers (Ying Qian and Na Li). Conflicts were resolved by reaching a consensus or arbitrated by a superior investigator (Yonghong Gao). The inclusion criteria were as follows: (1) full-text in Chinese or English; (2) AIS patients; (3) clearly defined inclusion criteria for AIS and HT; (4) comparison of UA concentrations between HT and non-HT patients; (5) studies that reporting UA concentrations as a continuous variable or categorical variable (≥3 equal categories); (6) human studies. The exclusion criteria were as follows: (1) incomplete or unavailable data; (2) conference abstracts, animal experiments, letters, comments, reviews, and case reports; and (3) in cases where data from the same population were presented in multiple studies, only the higher-quality study or the study with the largest sample size was included.

### Data extraction and quality assessment

Relevant parameters were independently extracted by two investigators (Ying Qian and Na Li) using a standardized data collection form. Disagreements were resolved through arbitration by a senior investigator (Yonghong Gao). The following data were abstracted: first author’s name, year, country, language, study design, number of patients, source of patients, mean age, gender, treatment, HT types, time to assessment; number of HT and non-HT patients, UA time point and detecting technique, UA concentrations (mean and standard deviation (mean ± SD), or median and interquartile range (IQR)), adjusted confounding factors, and conclusions. The Newcastle-Ottawa Scale (NOS) (30) was employed to assess the quality of each study. According to NOS, articles with a grade of 9 stars were considered to be of the highest quality, while those with a grade higher than 6 stars were considered to be of excellent quality.

### Statistical analysis

The STATA software (version 16.0, Stata Corp., College Station, TX) was utilized for statistical analysis. UA concentrations were reported as median, and IQR was converted to mean ± SD (31, 32). For data expressed as mean ± SD, the standardized mean difference (SMD) and 95% confidence interval (CI) were calculated for each study. Next, the computed result was combined and evaluated using either the random-effects model or the fixed-effects model. Besides, the Galbraith plot (33, 34) was used to assess heterogeneity across the studies. Sensitivity analyses were conducted to assess the robustness of the results. The Begg and Egger tests were used to evaluate publication bias, with *p* < 0.05 indicating the presence of publication bias.

## Results

### Study selection

**Figure 1:**
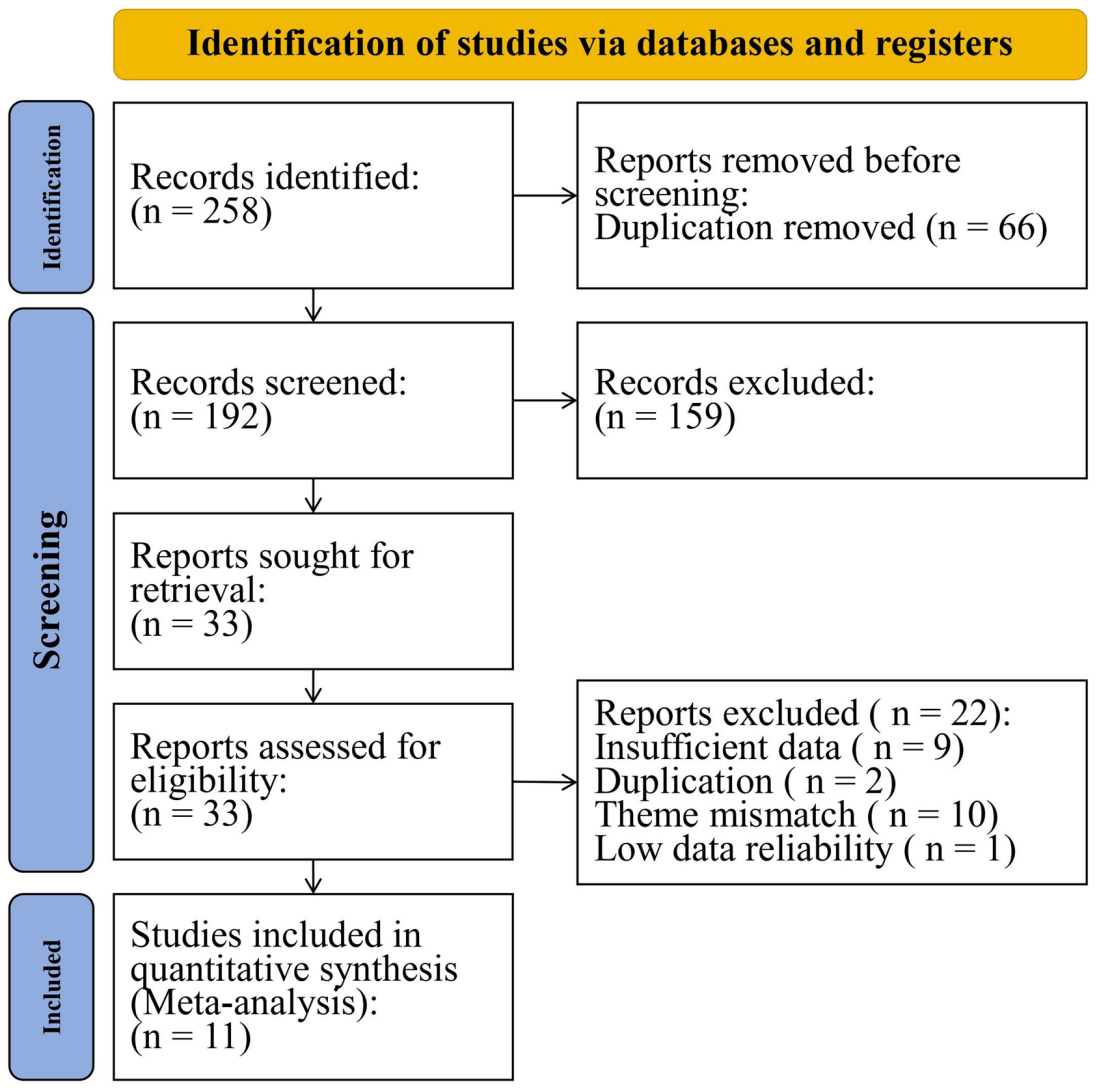
Flow diagram of study retrieval and screening.

As illustrated in Figure 1, the study selection process was based on the Preferred Reporting Item for Systematic Reviews and Meta-Analyses (PRISMA) principles (35). A total of 258 articles were retrieved from the databases, following which 66 duplicates were excluded. By reading the title and abstract of the remaining articles, 159 articles that did not meet the inclusion criteria were further excluded. Then, the retained 33 articles underwent full-text review. And 22 articles were excluded due to incomplete information (*n* = 9), duplication (*n* = 2), inconsistency with the research (*n* = 10), and low data reliability (*n* = 1). Finally, 11 articles of high quality were included in the meta-analysis (36–46).

### Study characteristics

The characteristics of the included studies are detailed in Table 1. All 11 studies included observational studies, among which 10 (36, 38–46) and 1 (37) were retrospective and prospective studies, respectively. Participants originated from China, with five and six articles published in Chinese (42–46) and English (36–41), respectively. The studies comprised 4,608 patients with AIS, including 701 patients with HT and 3,907 patients without HT. Interventions encompassed IVT (*n* = 6, 36, 39, 43–46) and EVT (*n* = 4, 37, 38, 41, 42), with one study not limiting IVT or EVT (40). Among the articles, 7 reported significantly higher UA concentrations in patients without HT compared to those with HT (36, 39–41, 43–45), with one article (36) demonstrating that irrespective of HT type, UA concentration was lower in HT patients than in non-HT patients. Interestingly, another article (39) identified lower UA concentration as an independent risk factor for HT in patients with large artery atherosclerosis stroke (LAA) patients or cardioembolism (CE). Meanwhile, an article (36) indicated that increasing UA concentrations are associated with favorable outcomes in AIS patients (345.67 ± 103.55 vs. 336.95 ± 95.5 μmol/L, *p* = 0.509). In addition, the optimal cutoff UA concentrations for differed across the 3 articles (36, 43, 45): 218.5 μmol/L, 284.00 μmol/L, 364.5 μmol/L. A study concluded that UA concentrations lower than 218.5 μmol/L or higher than 404.76 μmol/L were associated with an elevated risk of HT. Conversely, another paper (38) revealed that elevated UA concentrations were not only risk factors but also predictors of SICH after EVT. Moreover, 3 articles (37, 42, 46) reported that while UA concentrations were numerically higher in the HT group compared to the non-HT group, the difference was not statistically significant (*p* > 0.05). A study (42) identified hyperuricemia as a risk factor in AIS patients, while another study (37) documented that baseline high UA concentrations may predict superior 90-day functional outcomes. There are six articles (37, 38, 40, 41, 43, 45) mentioned UA concentration was measured by standard laboratory procedures with urate oxidase methods. All studies clearly reported their diagnostic methods for AIS and HT.

**Table 1:** Characteristics of the included studies.

First name, year	Country Language	Design	N M/F	Age (Mean ± SD)	Therapy	HT types	HT timepoint	UA timepoint	UA detecting technique	UA cutoff (μmol/L)	Segmentation of UA levels (μmol/L)	Case		UA (μmol/L)		Association between UA and HT	NOS
												HT	non-HT	HT	non-HT		
Zhang, 2021	China Chinese	Re	40 28/12	70.5 ± 6.5	IVT	HT	24 h after IVT	On admission	NR	NR	NR	11	29	294.7 ± 37.4	348.5 ± 63.5	Positive	5
Chen, 2021	China Chinese	Re	258 147/111	61.37 ± 12.36	EVT	HT	12 h、24 h、48 h after EVT	On admission	NR	NR	NR	62	196	311.25 ± 64.03	309.89 ± 57.16	No significant association	6
Sun, 2021	China Chinese	Re	228 167/61	69.32 ± 7.17	IVT	HT	24 h after IVT	On admission	oxidase assay	284.00	NR	30	198	281.20 ± 77.79	340.63 ± 78.95	Positive	6
Chen, 2020	China Chinese	Re	173 102/71	66.38 ± 8.72	IVT	HI PH	24 h after IVT	On admission	Automatic Biochemical Analyzer	>364.5	NR	46	127	324.89 ± 70.43	383.08 ± 89.21	Positive	8
Wei, 2017	China Chinese	Re	67 45/22	HT:73.25 ± 8.87 nHT:66.88 ± 10.28 68.40 ± 10.27	IVT	HT	72 h after IVT	On admission	NR	NR	NR	16	51	328.29 ± 49.48	306.86 ± 63.70	No significant association	4
Tian, Y., 2022	China English	Re	727 509/218	64	IVT	HI1; HI2; PH1; PH2	7 days after admission	48 h after IVT	NR	218.50	Q1 (<240.6) Q2 (240.6–302) Q3 (302–360) Q4 (>360)	112	615	253.65 ± 97.75	315.97 ± 96.42	Positive	8
Bai, H., 2022	China English	Pro	780 517/263	64.40 ± 12.14	EVT	sICH	24 h after EVT	24 h after EVT	standard laboratory procedures	NR	Q1 (<245.85) Q2 (245.85–297.45) Q3 (297.45–364.95) Q4 (≥364.95)	47	733	319.72 ± 120.67	309.39 ± 94.51	No significant association	8
Yang, C., 2020	China English	Re	247 177/70	63.2 ± 12.4	IVT	HI1; HI2; PH1; PH2; rPH	72 h after IVT	At 6 AM the day after admission.	NR	NR	NR	62	185	317.61 ± 87.45	346.62 ± 97.25	Positive; No significance in multivariate logistic regression analysis	7
Chen, Z., 2020	China English	Re	247 180/67	63.07 ± 12.60	EVT	HT sICH	72 h after admission	at 6 AM on the following day	OLYMPUS AU5400 automatic biochemical analyzer	NR	Q1 (<271) Q2 (271–338) Q3 (339–403) Q4 ((≥404)	92	155	322.60 ± 94.49	350.25 ± 99.28	Positive	7
Yuan, K., 2020	China English	Re	611 366/245	64.7 ± 12.3	EVT	sICH	NR	24 h after admission	Urate oxidase reagent on a Dax analyzer	NR	Q1 (<247.2) Q2 (247.2–310.0) Q3 (310.0–380.8) Q4 (≥380.8)	90	521	341.0(291.1–391.0) 341.04 ± 75.27	302.0(238.5–376.7) 305.92 ± 102.74	Higher UA level is risk factor of sICH	8
Song, Q., 2019	China English	Re	1,230 781/449	64.1 ± 14.5	IVT or EVT	HI, PH; sHT	7 days after admission	24 h after admission	Enzymatic methods; Roche Cobas C701	NR	Q1 (≤ 292.0) Q2 (292.1–377.0) Q3 (≥ 377.1)	133	1,097	272.1 ± 70.9	350.9 ± 105.6	Positive	8

Re, retrospective; Pro, prospective; N, number of patients; M/F, male/female; HT, hemorrhage transformation; non-HT, non-hemorrhage transformation; SD, standard deviation; IVT, intravenous thrombolysis; EVT, endovascular thrombectomy; HI, hemorrhagic infarction; PH, parenchymal hematoma; rPH, remote parenchymal hematoma; sICH, symptomatic intracranial hemorrhage; sHT, symptomatic hemorrhagic transformation; NR, not reported; UA, uric acid; Q, quartile of uric acid.

### Study quality

Study quality was evaluated using NOS criteria, as presented in Table 1. The results indicated good quality among the included publications, including 7 studies rated as good quality and 4 papers rated as suboptimal quality.

### Meta-analysis results

**Figure 2:**
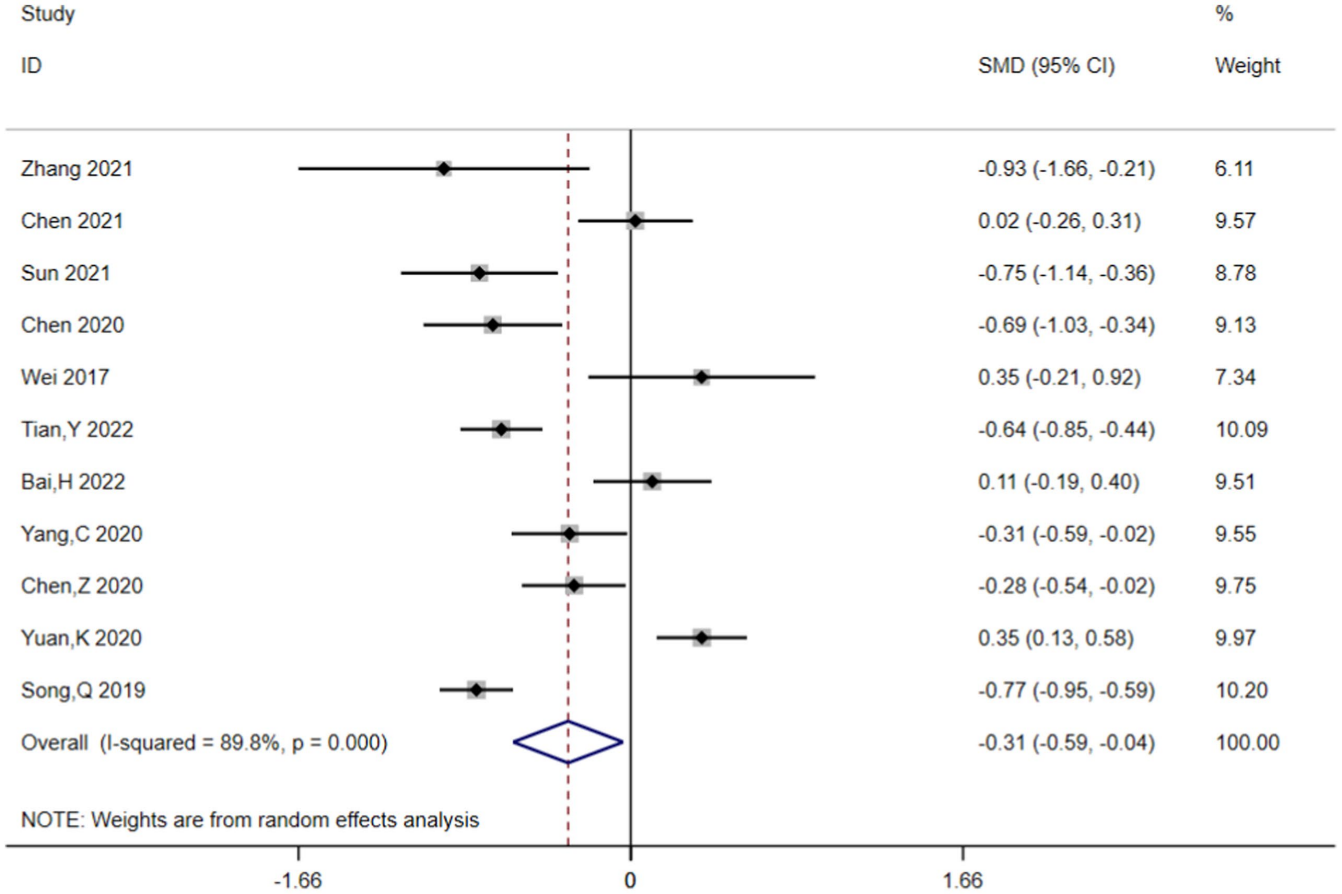
Lower UA concentrations were linked with higher HT after AIS.

The pooled SMDs suggested that compared with higher UA concentrations, lower UA concentrations were associated with HT after AIS (SMD = −0.313, 95% CI = −0.586–-0.039, *p* = 0.025) (Figure 2). However, heterogeneity (I^2^ = 89.8%, *p* < 0.001) was high across the studies. Consequently, the random-effects model was applied to pooled SMDs.

### Post- outlier analysis

**Figure 3:**
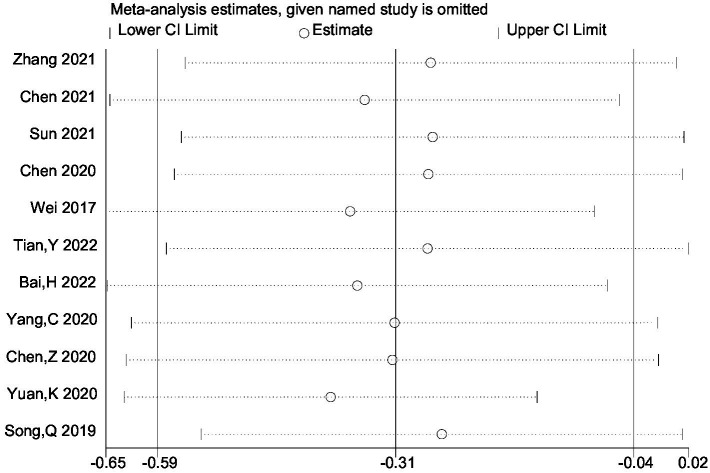
The provided study was taken away from the forest plot in the current meta-analysis’s sensitivity analysis.

**Figure 4:**
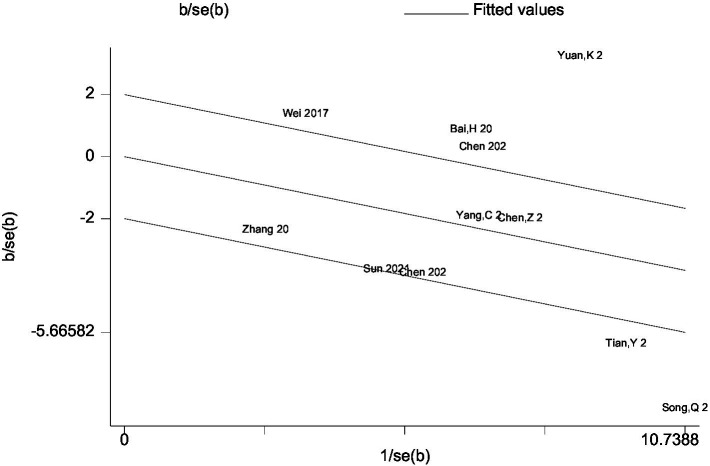
Galbraith plot analysis for outlier studies.

**Figure 5:**
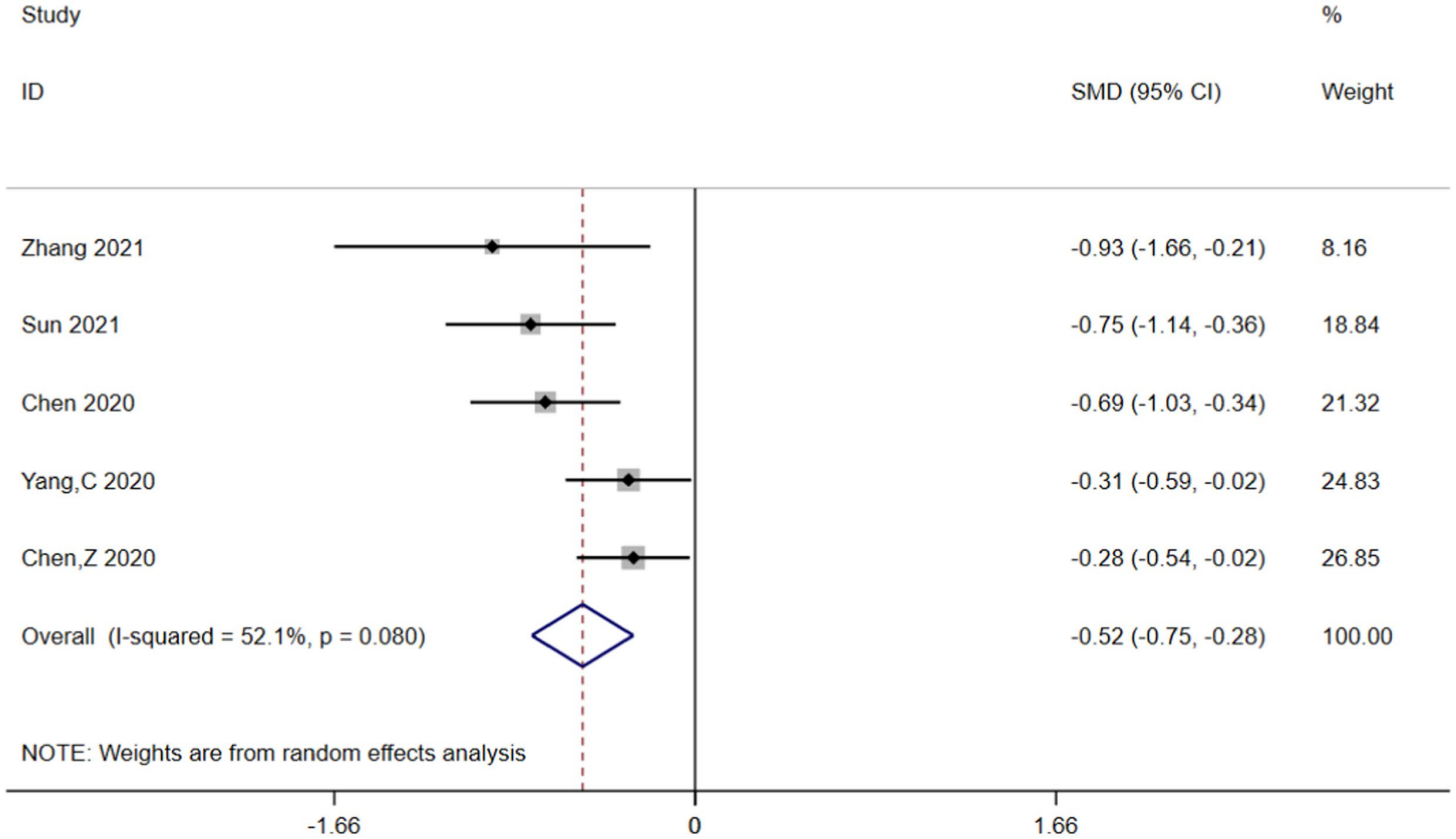
Forest plot analysis for the post-outlier outcomes indicated the UA concentrations in the HT group were lower compared with the non-HT group.

Heterogeneity was high among the trials (I^2^ = 89.8%, *p* < 0.001), prompting further investigation via sensitivity analysis (Figure 3) and the Galbraith plot (Figure 4). Sensitivity analyses were performed using the leave-one-out approach to examine the robustness of the results of the meta-analysis revealing consistent results in the pooled SMD analysis. The Galbraith plot identified several studies as outliers contributing to heterogeneity. As displayed in the Galbraith plot, half of the studies fell outside the regression line. However, excluding the outlier studies and reconducting forest plot analysis demonstrated the absence of heterogeneity in the remaining five studies (I^2^ = 52.1%, *p* = 0.080) (Figure 5), demonstrating that these outliers influenced heterogeneity. After adjusting for outliers, analysis using the random-effects model analysis outcomes (SMD = −0.517, 95% CI = −0.748–-0.285, *p* = 0.000) exposed that lower UA concentrations in the HT group compared with the non-HT group.

### Publication bias

**Figure 6:**
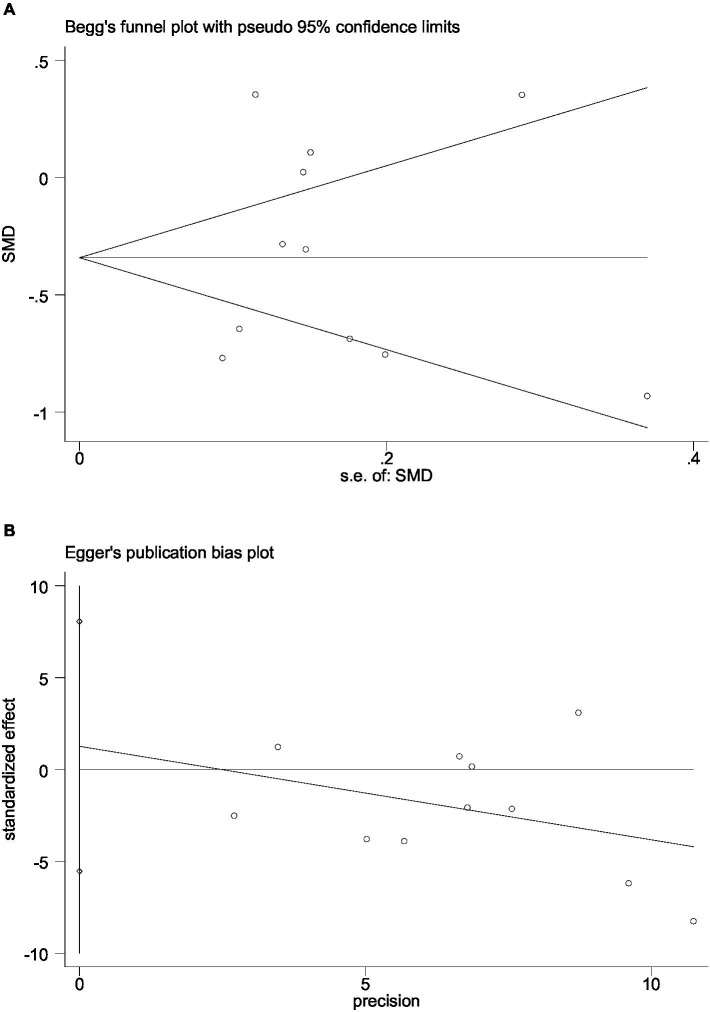
The funnel plot and Egger’s bias plot of publication bias in pooled SMDs analysis of all studies **(A,B)**.

Begg’s funnel plot and Egger’s test were utilized to assess publication bias among the 11 articles. Although Begg’s funnel plot (Figure 6A) was not completely symmetrical, the results of Egger’s test (Figure 6B) signaled the absence of publication bias in this meta-analysis, implying that the conclusions were reliable.

## Discussion

This meta-analysis included 11 articles involving 4,608 cases and examined the association between UA concentrations and the risk of HT in AIS patients. After adjusting for relevant covariates, our results demonstrated that lower UA concentrations were independently associated with an increased risk of HT in patients with AIS. The included articles all originated from China and included patients with AIS who underwent either IVT or EVT, with the primary outcome being the incidence of HT. According to the neuroimaging results, patients were divided into the HT and non-HT groups, and the mean ± SD or IQR values of UA concentrations were calculated for each group. The forest plot of pooled SMDs delineated that, compared with high UA concentrations, low UA concentrations were associated with a higher risk of HT following AIS (SMD = −0.313, 95% CI = −0.586–−0.039, *p* = 0.025). Meanwhile, heterogeneity was high among the studies (I^2^ = 89.8%, *p* < 0.001). Initially, subgroup analyses were performed based on language (Chinese or English), sample size (≤250 or more), age (≤65 or older), treatment (IVT or EVT), timing of HT assessment, timing of UA concentration measurements, and adjusted OR (YES or NO) to explore potential sources of heterogeneity, but heterogeneity remained high across groups (Figures not shown). Therefore, sensitivity analysis was performed, and the Galbraith plot was constructed to assess heterogeneity. The reliability of the results of the meta-analysis was demonstrated by sensitivity analysis using the leave-one-out approach. At the same time, Galbraith plot analysis identified six studies as potential sources of heterogeneity. Upon excluding these outliers, a subsequent forest plot of the remaining studies delineated a significant decrease in I^2^ value (I^2^ = 52.1%, *p* = 0.080). Notably, the re-evaluated pooled SMDs (SMD = −0.517, 95% CI = −0.748–−0.285, *p* = 0.000) still suggested that UA concentrations were lower in the HT group compared to the non-HT group. Moreover, Begg’s funnel plot and Egger’s test also showed the absence of publication bias in the meta-analysis. The above analysis collectively enhanced the credibility of our results.

Numerous studies have established the dual nature of UA. As is well-documented, UA accumulation drives the deposition of monosodium urate (MSU) crystals in the kidneys and joints, eventually leading to nephrolithiasis and gout. Epidemiological research has identified a correlation between elevated UA concentrations and hypertension, cardiovascular and cerebrovascular events, insulin resistance, and diabetes mellitus. Nicotinamide adenine dinucleotide phosphate-oxidase (NADPH) is activated by UA, which acts as a pro-oxidant in the cellular microenvironment and promotes oxidative stress. Additionally, UA can trigger inflammatory responses by releasing chemokines, and inflammatory markers and activating vasoconstrictive mediators, such as thromboxane, endothelin-1, and angiotensin II. Hyperuricemia limits NO bioavailability, thereby causing endothelial dysfunction, which can be attenuated via the administration of urinary acid-lowering drugs. In addition, UA also activates the renin-angiotensin system (RAS), thereby stimulating vascular smooth muscle cell proliferation and arterial stiffness (47–49). Numerous studies (22, 25) have evinced that UA can serve as an independent predictor of early death in AIS patients. Therefore, high UA concentrations may exacerbate diseases rather than contribute to clinical prognosis. Of note, four of the included studies showed that UA concentrations were higher in the HT group than in the non-HT group, with only one reaching statistical significance (*p* < 0.05). The study suggested that increasing UA concentrations not only increased the risk of SICH after EVT but also served as a predictor of SICH (38).

Recently, UA has garnered extensive attention as a potential neuroprotective agent against stroke (50), with a large number of studies investigating its anti-oxidant properties. As an endogenous extracellular antioxidant synthesized via purine metabolism, UA accounts for approximately 70% of total antioxidant capacity (51). It can inhibit the accumulation of reactive oxygen species and lipid peroxidation after exposure to glutamic acid or cyanide and scavenge free radicals generated during ischemia–reperfusion injury, thus exerting neuroprotective effects. A comprehensive review and meta-analysis (52) of the effects of UA in animal models of IS pointed out that elevated UA concentrations after IS can assist in reducing infarct size, improving BBB integrity, and enhancing neurological function. Meanwhile, a recent clinical meta-analysis (19) involving 10 eligible trials and including 8,131 AIS patients inferred that UA possessed protective effects on neurological outcomes in AIS patients. Furthermore, ascribed to its neuroprotective effect and significant decrease after stroke onset (47, 53), it has been explored as a clinical treatment for stroke during the past decades (54). A double-blind, randomized, vehicle-controlled study (55) found that UA can improve the prognosis of stroke by reducing the level of MMP-9 and alleviating oxidative stress. The URICO-ICTUS study (56) pointed out that the addition of UA to thrombolytic treatment yielded similar outcomes to placebo in stroke patients but did not elevate safety concerns.

At present, the relationship between UA concentrations and the risk of HT in AIS patients remains elusive. This meta-analysis showed that high UA concentrations lowered the risk of HT in AIS patients and concurrently improved their prognosis. Despite the pathogenesis of HT after AIS being unclear, studies (28, 36, 40, 57) hypothesized that ischemia–reperfusion injury (IRI) activates free radicals, thereby increasing the levels of reactive oxygen species (ROS) and reactive nitrogen species (RNS). The former promotes glutamate release, calcium overload, and neurotoxicity. Besides, it induces cell necrosis and apoptosis by activating adhesion molecules, promoting leukocyte infiltration, and releasing various cytokines. High concentrations of RNS can induce matrix metalloproteinase (MMP) activation, mediate BBB damage, expand infarct volume, and promote inflammation and apoptosis. The release of a large number of free radicals elicits oxidative stress, which in turn activates xanthine oxidase and increases the levels of endogenous urea, thereby inhibiting the activity of peroxynitrite and mitigating neuronal damage. As a neuroprotectant, UA can also suppress ischemia-induced inflammatory reactions, attenuate vascular impairment, preserve the integrity of the BBB, and decrease the infarct area by limiting the generation of reactive oxygen species on the blood vessel walls and decreasing MMP-9 activity.

This meta-analysis identified UA as a protective factor in patients with AIS. Specifically, patients with high UA concentrations were less likely to develop HT. In the enrolled studies, the optimal cutoff values for UA in HT patients were 218.5 μmol/L (36), 284 μmol/L (43), and 364.5 μmol/L (45), respectively. An article reported a normal UA range between 218.5 μmol/L and 404.76 μmol/L (36). Meanwhile, several extraneous factors, including the timing of blood collection, the use of diverse biochemical analyzers, individual variations, and regional differences, may have influenced the UA cutoff value for HT. Considering that existing studies have not established an optimal range value for UA, further large-scale standardized clinical studies are warranted in the future.

UA concentrations differed according to gender, and studies have confirmed that the average UA concentration is significantly lower in females than in males due to the effects of estrogen (40, 58). A sex-stratified analysis demonstrated that (40), UA concentrations were significantly higher in males than in females; correspondingly, the incidence of HT was lower in males than in females, consistent with the findings of earlier studies. This supported our conclusion that UA is a protective factor against HT post-AIS.

The substantial heterogeneity in this meta-analysis was not decreased by excluding individual studies. Differences in study design, endpoints, and duration of follow-up are potential sources of heterogeneity. Due to the high heterogeneity, the results were not sufficiently robust and should be interpreted with caution.

Nevertheless, several limitations of our study should not be overlooked. To begin, heterogeneity was high in this meta-analysis. Indeed, the Galbraith plot identifies six studies as potential sources of heterogeneity. Due to the lack of or incomplete information in these studies, the causes of the high heterogeneity could not be determined. Secondly, given that UA concentrations were extracted as a continuous variable without corresponding baseline levels, heterogeneity was high, and the possibility of confounding factors compromising the results cannot be neglected. Other risk factors, such as a history of hypertension and atrial fibrillation mentioned in the included articles, should not be ignored. Thirdly, each included study exclusively measured UA concentrations once without accounting for dynamic fluctuations in UA concentrations during the course of AIS. Therefore, additional studies are necessary to measure UA values multiple times to dynamically monitor the relationship between UA concentrations and the risk of HT. Fourthly, subgroup analyses were not performed on AIS type, HT type, or gender owing to limited data. Further clinical studies targeting relevant subgroups should be conducted in the future. Eventually, all the studies were conducted in China, limiting the applicability of our results to a broader population.

## Conclusion

This meta-analysis highlighted that UA exerts neuroprotective effects in AIS patients and that lower UA concentrations may increase the risk of HT following AIS, thereby laying a theoretical reference for future related studies. At the same time, several challenges also need to be addressed in the future, such as monitoring dynamic UA concentrations and identifying the optimal range for UA.

## Data availability statement

The original contributions presented in the study are included in the article/supplementary material, further inquiries can be directed to the corresponding author.

## Author contributions

YQ: Data curation, Methodology, Writing – original draft, Writing – review & editing. NL: Writing – original draft, Writing – review & editing. YL: Data curation, Investigation, Writing – review & editing. CT: Formal analysis, Investigation, Software, Writing – review & editing. ZL: Formal analysis, Investigation, Writing – review & editing. GZ: Data curation, Formal analysis, Methodology, Writing – review & editing. FY: Resources, Software, Writing – original draft. HZ: Data curation, Software, Writing – original draft. YG: Funding acquisition, Project administration, Supervision, Writing – review & editing.
